# The association between TMAO, CMPF, and clinical outcomes in advanced chronic kidney disease: results from the European QUALity (EQUAL) Study

**DOI:** 10.1093/ajcn/nqac278

**Published:** 2022-09-27

**Authors:** Lu Dai, Ziad A Massy, Peter Stenvinkel, Nicholas C Chesnaye, Islam Amine Larabi, Jean Claude Alvarez, Fergus J Caskey, Claudia Torino, Gaetana Porto, Maciej Szymczak, Magdalena Krajewska, Christiane Drechsler, Christoph Wanner, Kitty J Jager, Friedo W Dekker, Pieter Evenepoel, Marie Evans, the EQUAL Study Investigators, Andreas Schneider, Anke Torp, Beate Iwig, Boris Perras, Christian Marx, Christiane Drechsler, Christof Blaser, Christoph Wanner, Claudia Emde, Detlef Krieter, Dunja Fuchs, Ellen Irmler, Eva Platen, Hans Schmidt-Gürtler, Hendrik Schlee, Holger Naujoks, Ines Schlee, Sabine Cäsar, Joachim Beige, Jochen Röthele, Justyna Mazur, Kai Hahn, Katja Blouin, Katrin Neumeier, Kirsten Anding-Rost, Lothar Schramm, Monika Hopf, Nadja Wuttke, Nikolaus Frischmuth, Pawlos Ichtiaris, Petra Kirste, Petra Schulz, Sabine Aign, Sandra Biribauer, Sherin Manan, Silke Röser, Stefan Heidenreich, Stephanie Palm, Susanne Schwedler, Sylke Delrieux, Sylvia Renker, Sylvia Schättel, Theresa Stephan, Thomas Schmiedeke, Thomas Weinreich, Til Leimbach, Torsten Stövesand, Udo Bahner, Wolfgang Seeger, Adamasco Cupisti, Adelia Sagliocca, Alberto Ferraro, Alessandra Mele, Alessandro Naticchia, Alex Còsaro, Andrea Ranghino, Andrea Stucchi, Angelo Pignataro, Antonella De Blasio, Antonello Pani, Aris Tsalouichos, Bellasi Antonio, Biagio Raffaele Di Iorio, Butti Alessandra, Cataldo Abaterusso, Chiara Somma, Claudia D'Alessandro, Claudia Torino, Claudia Zullo, Claudio Pozzi, Daniela Bergamo, Daniele Ciurlino, Daria Motta, Domenico Russo, Enrico Favaro, Federica Vigotti, Ferruccio Ansali, Ferruccio Conte, Francesca Cianciotta, Francesca Giacchino, Francesco Cappellaio, Francesco Pizzarelli, Gaetano Greco, Gaetana Porto, Giada Bigatti, Giancarlo Marinangeli, Gianfranca Cabiddu, Giordano Fumagalli, Giorgia Caloro, Giorgina Piccoli, Giovanbattista Capasso, Giovanni Gambaro, Giuliana Tognarelli, Giuseppe Bonforte, Giuseppe Conte, Giuseppe Toscano, Goffredo Del Rosso, Irene Capizzi, Ivano Baragetti, Lamberto Oldrizzi, Loreto Gesualdo, Luigi Biancone, Manuela Magnano, Marco Ricardi, Maria Di Bari, Maria Laudato, Maria Luisa Sirico, Martina Ferraresi, Michele Provenzano, Moreno Malaguti, Nicola Palmieri, Paola Murrone, Pietro Cirillo, Pietro Dattolo, Pina Acampora, Rita Nigro, Roberto Boero, Roberto Scarpioni, Rosa Sicoli, Rosella Malandra, Silvana Savoldi, Silvio Bertoli, Silvio Borrelli, Stefania Maxia, Stefano Maffei, Stefano Mangano, Teresa Cicchetti, Tiziana Rappa, Valentina Palazzo, Walter De Simone, Anita Schrander, Bastiaan van Dam, Carl Siegert, Carlo Gaillard, Charles Beerenhout, Cornelis Verburgh, Cynthia Janmaat, Ellen Hoogeveen, Ewout Hoorn, Friedo Dekker, Johannes Boots, Henk Boom, Jan-Willem Eijgenraam, Jeroen Kooman, Joris Rotmans, Kitty Jager, Liffert Vogt, Maarten Raasveld, Marc Vervloet, Marjolijn van Buren, Merel van Diepen, Nicholas Chesnaye, Paul Leurs, Pauline Voskamp, Sadie van Esch, Siska Boorsma, Stefan Berger, Constantijn Konings, Zeynep Aydin, Aleksandra Musiała, Anna Szymczak, Ewelina Olczyk, Hanna Augustyniak-Bartosik, Ilona Miśkowiec-Wiśniewska, Jacek Manitius, Joanna Pondel, Kamila Jędrzejak, Katarzyna Nowańska, Łukasz Nowak, Maciej Szymczak, Magdalena Durlik, Szyszkowska Dorota, Teresa Nieszporek, Zbigniew Heleniak, Andreas Jonsson, Björn Rogland, Carin Wallquist, Denes Vargas, Emöke Dimény, Fredrik Sundelin, Fredrik Uhlin, Gunilla Welander, Isabel Bascaran Hernandez, Knut-Christian Gröntoft, Maria Stendahl, Maria Eriksson Svensson, Marie Evans, Olof Heimburger, Pavlos Kashioulis, Stefan Melander, Tora Almquist, Alistair Woodman, Anna McKeever, Asad Ullah, Barbara McLaren, Camille Harron, Carla Barrett, Charlotte O'Toole, Christina Summersgill, Colin Geddes, Deborah Glowski, Deborah McGlynn, Dympna Sands, Fergus Caskey, Geena Roy, Gillian Hirst, Hayley King, Helen McNally, Houda Masri-Senghor, Hugh Murtagh, Hugh Rayner, Jane Turner, Joanne Wilcox, Jocelyn Berdeprado, Jonathan Wong, Joyce Banda, Kirsteen Jones, Lesley Haydock, Lily Wilkinson, Margaret Carmody, Maria Weetman, Martin Joinson, Mary Dutton, Michael Matthews, Neal Morgan, Nina Bleakley, Paul Cockwell, Paul Roderick, Phil Mason, Philip Kalra, Rincy Sajith, Sally Chapman, Santee Navjee, Sarah Crosbie, Sharon Brown, Sheila Tickle, Suresh Mathavakkannan, Ying Kuan

**Affiliations:** Aging Research Center, Department of Neurobiology, Care Sciences and Society, Karolinska Institutet, Stockholm, Sweden; Division of Renal Medicine, Department of Clinical Science, Intervention and Technology, Karolinska Institutet, Stockholm, Sweden; Division of Nephrology, Ambroise Paré University Hospital, Boulogne-Billancourt, France; Centre for Research in Epidemiology and Population Health (CESP), Inserm UMRS 1018, Team 5, University Versailles-Saint Quentin, University Paris-Saclay, Paris, France; Division of Renal Medicine, Department of Clinical Science, Intervention and Technology, Karolinska Institutet, Stockholm, Sweden; ERA-EDTA Registry, Department of Medical Informatics, Academic Medical Center, University of Amsterdam, Amsterdam Public Health Research Institute, Amsterdam, The Netherlands; Laboratory of Pharmacology and Toxicology, CHU, Raymond Poincare, Garches, France; INSERM U1173, UFR des Sciences de la Santé Simone Veil, Montigny le Bretonneux, Université de Versailles-Saint-Quentin-en-Yvelines, Versailles, France; Laboratory of Pharmacology and Toxicology, CHU, Raymond Poincare, Garches, France; INSERM U1173, UFR des Sciences de la Santé Simone Veil, Montigny le Bretonneux, Université de Versailles-Saint-Quentin-en-Yvelines, Versailles, France; Population Health Sciences, Bristol Medical School, University of Bristol, Bristol, United Kingdom; IFC-CNR, Clinical Epidemiology and Pathophysiology of Renal Diseases and Hypertension, Reggio Calabria, Italy; G.O.M., Bianchi Melacrino Morelli, Reggio Calabria, Italy; Clinical Department of Nephrology and Transplantation Medicine, Wroclaw Medical University, Wroclaw, Poland; Clinical Department of Nephrology and Transplantation Medicine, Wroclaw Medical University, Wroclaw, Poland; Division of Nephrology, University Hospital of Würzburg, Würzburg, Germany; Division of Nephrology, University Hospital of Würzburg, Würzburg, Germany; ERA-EDTA Registry, Department of Medical Informatics, Academic Medical Center, University of Amsterdam, Amsterdam Public Health Research Institute, Amsterdam, The Netherlands; ERA-EDTA Registry, Department of Medical Informatics, Academic Medical Center, University of Amsterdam, Amsterdam Public Health Research Institute, Amsterdam, The Netherlands; Department of Microbiology, Immunology, and Transplantation, Nephrology and Renal Transplantation Research Group, KU Leuven, Leuven, Belgium; Department of Nephrology and Renal Transplantation, University Hospitals Leuven, Leuven, Belgium; Division of Renal Medicine, Department of Clinical Science, Intervention and Technology, Karolinska Institutet, Stockholm, Sweden

**Keywords:** uremic toxins, trimethylamine N-oxide, 3-carboxy-4-methyl-5-propyl-2-furanpropionate, red meat, fish intake, CKD, mortality, kidney replacement therapy

## Abstract

**Background:**

Trimethylamine N-oxide (TMAO), a metabolite from red meat and fish consumption, plays a role in promoting cardiovascular events. However, data regarding TMAO and its impact on clinical outcomes are inconclusive, possibly due to its undetermined dietary source.

**Objectives:**

We hypothesized that circulating TMAO derived from fish intake might cause less harm compared with red meat sources by examining the concomitant level of 3-carboxy-4-methyl-5-propyl-2-furanpropionate (CMPF), a known biomarker of fish intake, and investigated the association between TMAO, CMPF, and outcomes.

**Methods:**

Patients were recruited from the European QUALity (EQUAL) Study on treatment in advanced chronic kidney disease among individuals aged ≥65 y whose estimated glomerular filtration rate (eGFR) had dropped for the first time to ≤20 mL/min per 1.73 m^2^ during the last 6 mo. The association between TMAO, CMPF, and outcomes including all-cause mortality and kidney replacement therapy (KRT) was assessed among 737 patients. Patients were further stratified by median cutoffs of TMAO and CMPF, suggesting high/low red meat and fish intake.

**Results:**

During a median of 39 mo of follow-up, 232 patients died. Higher TMAO was independently associated with an increased risk of all-cause mortality (multivariable HR: 1.46; 95% CI: 1.17, 1.83). Higher CMPF was associated with a reduced risk of both all-cause mortality (HR: 0.79; 95% CI: 0.71, 0.89) and KRT (HR: 0.80; 95% CI: 0.71, 0.90), independently of TMAO and other clinically relevant confounders. In comparison to patients with low TMAO and CMPF, patients with low TMAO and high CMPF had reduced risk of all-cause mortality (adjusted HR: 0.49; 95% CI: 0.31, 0.73), whereas those with high TMAO and high CMPF showed no association across adjusted models.

**Conclusions:**

High CMPF conferred an independent role in health benefits and might even counteract the unfavorable association between TMAO and outcomes. Whether higher circulating CMPF concentrations are due to fish consumption, and/or if CMPF is a protective factor, remains to be verified.

## Introduction

Patients with chronic kidney disease (CKD) are characterized with a high prevalence of cardiovascular (CV) comorbidities and increased risk of mortality (1). Traditional risk factors such as old age, diabetes, hypertension, and hyperlipidemia are only partly accountable for the excessive CV burden and adverse clinical outcomes (2, 3), suggesting other residual factors as culprits in the setting of CKD. Recent research has highlighted the role of diet and gut microbiota in mediating the generation and metabolism of some uremic toxins, as well as the consequent impact on host health (4–8).

One of the most representative uremic toxins linking diet, gut microbiome, and host health is trimethylamine N-oxide (TMAO). Although multiple studies have reported the link between TMAO and risk of cardiometabolic diseases (9–11), the clinical evidence linking TMAO to adverse CV outcomes are discordant (12–18). Another uremic toxin is 3-carboxycarboxy-4-methyl-5-propyl-2-furanpropionate (CMPF), a metabolite of furan fatty acids (19, 20). Despite being generally considered as a protein-bound (>95%) uremic toxin highly elevated in advanced CKD (21, 22), the prognostic role of CMPF in CKD is scarcely explored. The richest dietary sources of furan fatty acids and their metabolite CMPF are fish and fish oils. Moreover, several studies have identified plasma CMPF as a biomarker for fish consumption (23). The main dietary source of TMAO, on the contrary, is animal food, including red meat and egg rich in choline and carnitine, but also fish. Although red meat and fish consumption can cause increased plasma or urine concentrations of TMAO (24, 25), this marker, in contrast to CMPF, has not yet been verified as a specific marker of red meat or fish intake.

We hypothesized that TMAO derived from fish consumption might cause less harm compared with TMAO from mainly red meat/egg. Given the elusive and divergent metabolic properties of TMAO and CMPF, we postulated that integrating these 2 uremic toxins, as representative metabolites of dietary intake of red meat and/or fish intake, may provide a better perspective to elucidate their role in health outcomes in the context of CKD. To fill this gap, we investigated the association between TMAO, CMPF, and clinical outcomes including risk of all-cause mortality and kidney replacement therapy (KRT) initiation in carefully phenotyped patients aged ≥65 y old with advanced CKD.

## Methods

### Study design

The European QUALity (EQUAL) Study on treatment in advanced chronic kidney disease is a prospective cohort study in advanced CKD patients in Germany, Italy, Poland, Sweden, the Netherlands, and the United Kingdom (26). Approval was obtained from the Medical Ethical Committees of the national coordinating centers and corresponding institutional review boards of the participating centers. Written informed consent was obtained from all patients. A full description of the study has been published elsewhere (26). Patients ≥65 y of age were included if their estimated glomerular filtration rate (eGFR), as estimated by the Modification of Diet in Renal Disease (MDRD) equation (27), had dropped for the first time to ≤20 mL/min per 1.73 m^2^ during the previous 6 mo. Patients were followed until kidney transplantation, death, moving to a center outside the designation of the EQUAL study, refusal of further participation, or loss to follow-up or end of follow-up, whichever came first. For the current analyses, we included patients with baseline measurement of TMAO and CMPF (between March 2012 and February 2019) and examined events of interest within a maximum of 48 mo follow-up (see flowchart in **Supplemental Figure 1**).

### Data collection

Patients’ clinical data from the baseline visit occurring within 6 mo from the index eGFR were entered in a web-based clinical record form, covering information on the patients’ demographics, primary kidney disease, comorbid conditions, medication, physical examination, and routine laboratory results. Data on preexisting cardiovascular disease (CVD), including cerebrovascular disease, peripheral vascular disease, myocardial infarction, angina pectoris, congestive heart failure, left ventricular hypertrophy, and cardiac arrhythmias, were recorded. The 7-point subjective global assessment (SGA) tool was used to assess patients’ nutritional status (28), and SGA <5 was defined as malnutrition.

### TMAO and CMPF

Upon enrollment of the patients, serum samples were collected and immediately frozen. The samples were thawed immediately prior to analysis. The investigated 2 uremic toxins (CMPF and TMAO) were assayed in serum samples collected at baseline using a validated ultra-performance LC–tandem MS (UPLC-MS/MS) technique (29). To determine total concentrations, serum samples were first precipitated with methanol. The supernatant was evaporated with nitrogen and then reconstituted in 80 µL of water. The assay's limit of quantification was between 10 and 50 ng/mL, depending on the compound. The intra- and interassay variabilities (evaluated at 3 different concentrations: 150, 8000, and 40,000 ng/mL) for the 2 compounds were all <13%.

### Outcomes

The primary outcomes were all-cause mortality and KRT initiation during 48 mo of follow-up. In a sensitivity analysis, we explored first major adverse cardiac events (MACEs) as an additional outcome. Information on outcomes was collected as a part of the study protocol at each planned study visit. The cause of death was assigned by the treating doctor and registered in the EQUAL study whenever it was available.

### Statistical analysis

Baseline characteristics are presented as means ± SDs for normally distributed continuous variables, medians with IQRs for skewed continuous variables, and categorical variables as percentages. Statistical significance was set at the level of *P* < 0.05. Comparisons between TMAO and CMPF tertile groups were assessed with the Kruskal-Wallis test for skewed continuous variables, 1-factor ANOVA for normally distributed variables, and the chi-square test for categorical variables. Multivariable regression analysis was used to investigate the association between TMAO, CMPF, and other clinically relevant parameters. Multiple imputation was used to minimize the risk of bias. Missing values at baseline [including Charlson comorbidity index, diastolic blood pressure (BP), systolic BP, height, weight, hemoglobin, albumin, phosphate, and albumin creatinine ratio (ACR)] were imputed using multivariable normal regression (20 repetitions). In regression analyses, continuous variables with nonnormal distributions were converted as natural log (ln)-transformed values. MACE was defined as a hospitalization or comorbidity due to cerebrovascular disease, myocardial infarction, peripheral vascular disease, angina pectoris, arrhythmias, coronary artery disease, or death due to myocardial ischemia and infarction, cardiac arrest, or cerebrovascular accident. First MACE refers to the first occurrence of an MACE after entering the study. We used Cox proportional hazards regression to examine unadjusted and multivariable adjusted associations between TMAO, CMPF, and risk of death and first MACE. We adjusted for demographic characteristics (age, sex, and country) in model 1, with further adjustment for traditional risk factors (diabetes, CVD, Charlson comorbidity index, diastolic BP, systolic BP, SGA) in model 2, and CKD-related biochemical parameters (albumin, phosphate, hemoglobin) in model 3 and eGFR in model 4. Schoenfeld residuals were examined to confirm the proportionality assumption. We applied cause-specific Cox proportional hazards regression models to evaluate the relation between TMAO, CMPF, and risk of KRT with death as a competing risk. The competing risk model was adjusted for conventional risk factors of dialysis (age, sex, eGFR, and ACR) in model 1, further adjusted for other confounders in model 2 (country, diabetes, CVD, Charlson comorbidity index, diastolic BP, systolic BP, SGA) and CKD-related biochemical parameters (albumin, phosphate, hemoglobin) in model 3. To test the assumption of TMAO and CMPF as biomarkers of red meat and fish intake and whether such a pattern is associated with clinical outcomes, we categorized patients into 4 groups according to the median cutoff medians of TMAO and CMPF: low TMAO (–) and low CMPF (–) (suggesting low red meat and low fish intake), low TMAO (–) and high CMPF (+) (suggesting low red meat and high fish intake), high TMAO (+) and low CMPF (–) (suggesting high red meat and low fish intake), and high TMAO (+) and high CMPF (+) (suggesting high red meat and/or fish intake). The association between categorized TMAO and CMPF subgroups and outcomes were tested in Cox regression models as mentioned previously. We repeated analyses both in original and imputed datasets, and for convenience, results derived from imputed datasets are presented. Statistical analyses were performed using Stata 16.1 (StataCorp).

## Results

### Baseline characteristics and TMAO and CMPF concentrations

The main characteristics of 737 patients with baseline serum TMAO and CMPF measurements are described in **Table 1**. The median age was 76 y, 63.1% were male, and eGFR at baseline was 19.7 (IQR: 16.0, 23.0) mL/min per 1.73 m^2^. Forty-six percent of patients had preexisting CVD, 43% had diabetes, and 32% showed signs of malnutrition (SGA <5). At baseline, 9% were prescribed a low-protein diet (<0.8 g ⸱ kg per day). The median serum TMAO was 18.2 (IQR: 11.9, 28.5) µM, and CMPF was 7.1 (IQR: 2.6, 14.9) µM. Patient characteristics according to the tertiles of TMAO and CMPF are presented in **Supplemental Tables 1** and **2**.

**TABLE 1:** Baseline characteristics of patients with baseline TMAO and CMPF measurements^1^

	Values
Age, y	76.0 (70.4, 80.8)
Male sex, *n* (%)	465 (63.1%)
Country, *n* (%)	
Germany	115 (15.6%)
Poland	61 (8.3%)
Sweden	213 (28.9%)
United Kingdom	348 (47.2%)
Primary renal diagnosis (*n* = 729), *n* (%)	
Glomerular disease	85 (11.5%)
Tubulo-interstitial disease	63 (8.5%)
Systemic disease affecting the kidney	21 (2.8%)
Diabetes	165 (22.4%)
Hypertension	232 (31.5%)
Familial/hereditary nephropathies	33 (4.5%)
Miscellaneous renal disorders	23 (3.1%)
Unknown	107 (14.5%)
Charlson comorbidity index (*n* = 726)	7 (6–8)
Previous CVD, *n* (%)	335 (45.5%)
Diabetes, *n* (%)	319 (43.3%)
eGFR (MDRD), mL/min per 1.73 m^2^	19.7 (16.0, 23.0)
Diastolic BP (*n* = 727), mmHg	74 (67, 81)
Systolic BP (*n* = 727), mmHg	147 (132, 160)
Height (*n* = 695), cm	169 (161, 175)
Weight (*n* = 712), kg	80.0 (69.9, 91.5)
BMI (*n* = 686), kg/m^2^	28.2 (24.9, 32.0)
BMI group (kg/m^2^) (*n* = 686), *n* (%)	
<22	49 (6.6%)
22–24.9	130 (17.6%)
25–29.9	250 (33.9%)
>30	257 (34.9%)
Waist circumference (*n* = 700), cm	103 (95, 113)
ɑ-Blocker, *n* (%)	204 (27.7%)
B-Blocker, *n* (%)	414 (56.2%)
ACEi/ARB, *n* (%)	385 (52.2%)
Lipid-lowering, *n* (%)	466 (63.2%)
SGA overall assessment	6.0 (5.0, 7.0)
SGA <5 (malnourished), *n* (%)	239 (32.4%)
Low-protein-diet prescription (*n* = 732), *n* (%)	68 (9.3%)
Hemoglobin (*n* = 715), mmol/L	7.1 (6.6, 7.9)
Sodium (*n* = 721), mmol/L	141.0 (139.0, 142.0)
Potassium (*n* = 724), mmol/L	4.6 (4.2, 5.0)
Calcium (*n* = 699), mmol/L	2.3 (2.2, 2.4)
Phosphate (*n* = 690), mmol/L	1.3 (1.1, 1.4)
Urea (*n* = 714), mmol/L	17.8 (14.6, 22.2)
Albumin (*n* = 657), g/L	38.0 (34.2, 41.0)
Total cholesterol (*n* = 550), mmol/L	4.5 (3.7, 5.5)
PTH (*n* = 587), pmol/L	15.4 (9.0, 23.5)
ACR (*n* = 335), mg/mmol	49.3 (8.5, 198.4)
TMAO, µM	18.2 (11.9–28.5)
CMPF, µM	7.1 (2.6, 14.9)

1 Values are medians (IQR), or *n* (%), as appropriate; *n* = 737. ACEi/ARB, angiotensin-converting enzyme inhibitor/angiotensin receptor blocker; ACR, albumin creatinine ratio; BP, blood pressure; CMPF, 3-carboxy-4-methyl-5-propyl-2-furanpropionate; CVD, cardiovascular disease; eGFR, estimated glomerular filtration rate; MDRD, Modification of Diet in Renal Disease; PTH, parathyroid hormone; SGA, subjective global assessment; TMAO, trimethylamine N-oxide.

### Association between TMAO, CMPF, and other clinical parameters

In age- and sex-adjusted multivariable regression analysis, while male sex (adjusted for age), diabetes, phosphate, and CMPF were positively associated with TMAO, hemoglobin and eGFR were negatively associated with TMAO (**Table 2**). Age, male sex, hemoglobin, height, and TMAO were positively associated with CMPF, whereas compared with patients recruited in Germany, patients from Sweden were associated with higher CMPF and patients from the United Kingdom were associated with lower CMPF concentrations (Table 2).

**TABLE 2:** Multivariable linear regression of factors associated with TMAO and CMPF^1^

	ln TMAO		ln CMPF	
	Coefficient (SE)	*P*	Coefficient (SE)	*P*
ln Age^2^	0.14 (0.28)	0.61	1.34 (0.49)	0.007
Male sex^3^	0.23 (0.05)	<0.0001	0.18 (0.09)	0.05
Country (Germany as reference group)				
Poland	-0.11 (0.11)	0.29	-0.01 (0.18)	0.96
Sweden	0.03 (0.08)	0.67	0.52 (0.13)	<0.0001
United Kingdom	-0.08 (0.72)	0.26	-0.37 (0.12)	0.002
Diabetes, yes vs. no	0.17 (0.05)	0.001	-0.18 (0.09)	0.04
Previous CVD, yes vs. no	-0.04 (0.05)	0.49	0.17 (0.09)	0.06
SGA <5, malnutrition	0.05 (0.05)	0.39	-0.12 (0.09)	0.20
Charlson comorbidity index	0.17 (0.10)	0.08	-0.29 (0.18)	0.10
ln Hemoglobin	-0.63 (0.19)	0.001	0.96 (0.33)	0.003
ln Albumin	-0.25 (0.12)	0.04	0.31(0.21)	0.14
ln Height	0.49 (0.61)	0.48	2.09 (1.07)	0.05
ln Phosphate	0.71 (0.12)	<0.0001	-0.32 (0.19)	0.11
ln eGFR	-0.73 (0.08)	<0.0001	0.25 (0.14)	0.08
ln CMPF	0.07 (0.02)	0.001	—	—
ln TMAO	—	—	0.23 (0.06)	0.001

1 *n* = 737. Each model is age- and sex-adjusted. CMPF, 3-carboxy-4-methyl-5-propyl-2-furanpropionate; CVD, cardiovascular disease; eGFR, estimated glomerular filtration rate; SGA, subjective global assessment; TMAO, trimethylamine N-oxide.

2 Model adjusted for sex.

3 Model adjusted for age.

### Association between TMAO, CMPF, and outcomes

#### TMAO, CMPF, and all-cause mortality

During a median of 39 mo of follow-up, 232 patients died and 258 patients initiated KRT. The unadjusted and adjusted HRs for all-cause mortality in Cox regression models are presented in **Table 3**. In the crude model, ln CMPF and ln TMAO were significant predictors of all-cause mortality [HRs: 0.81 (95% CI: 0.73, 0.90) and 1.63 (95% CI: 1.33, 2.00), respectively]. These associations remained statistically significant after extensive adjustments across models (Table 3).

**TABLE 3:** Association between TMAO, CMPF, and risk of all-cause mortality^1^

	All-cause mortality, HR [95% CI]	
	ln CMPF	ln TMAO
Crude	0.81 [0.73,0.90]	1.63 [1.33,2.00]
Model 1	0.76 [0.68,0.84]	1.63 [1.32,2.00]
Model 2	0.78 [0.70,0.87]	1.55 [1.26,1.91]
Model 3	0.79 [0.70,0.88]	1.51 [1.23,1.87]
Model 4	0.79 [0.71,0.89]	1.46 [1.17,1.83]

1 *n* = 737. Crude model: ln TMAO + ln CMPF, Model 1: adjusted for age, sex, and country. Model 2: model 1 + diabetes, preexisting cardiovascular disease, Charlson comorbidity index, diastolic blood pressure, systolic blood pressure, and malnutrition (SGA <5). Model 3: model 2 + albumin, phosphate, and hemoglobin. Model 4: model 3 + eGFR. CMPF, 3-carboxy-4-methyl-5-propyl-2-furanpropionate; eGFR, estimated glomerular filtration rate; SGA, subjective global assessment; TMAO, trimethylamine N-oxide.

**FIGURE 1:**
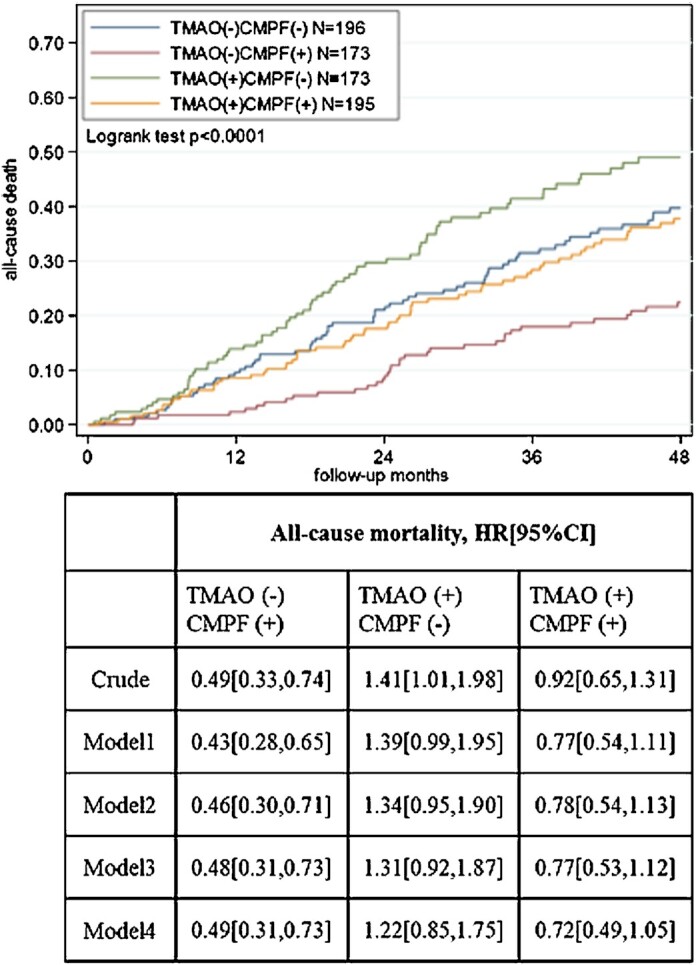
Kaplan–Meier estimates for all-cause mortality and cause-specific Cox regression models according to TMAO and CMPF median groups. Model 1 adjusted for age, sex, country. Model 2: adjusted as for model 1 plus diabetes, preexisting cardiovascular disease, Charlson comorbidity index, diastolic blood pressure, systolic blood pressure, malnutrition (SGA <5). Model 3: adjusted as for model 2 plus albumin, phosphate, hemoglobin. Model 4: adjusted as for model 3 plus eGFR. CMPF, 3-carboxy-4-methyl-5-propyl-2-furanpropionate; eGFR, estimated glomerular filtration rate; SGA, subjective global assessment; TMAO, trimethylamine N-oxide.

The association between TMAO and CMPF subgroups and all-cause mortality is presented in **Figure 1**. In the crude model, in comparison to patients with TMAO (–) CMPF (–) (as reference group), the subgroup with TMAO (–) CMPF (+) had substantially lower risk of all-cause mortality (HR: 0.49; 95% CI: 0.33, 0.74). The significant lower mortality risk for the TMAO (–) CMPF (+) patient group (51–57% lower risk) was sustained after multivariable adjustments. In contrast, patients with TMAO (+) CMPF (–) showed a higher risk of mortality only in a crude model (HR: 1.41; 95% CI: 1.01, 1.98), whereas patients with TMAO (+) CMPF (+) showed no association with risk of mortality across crude and adjusted models.

#### TMAO, CMPF, and risk of KRT initiation

In the unadjusted crude model, ln CMPF and ln TMAO were predictors of risk of KRT [HRs: 0.74 (95% CI: 0.67, 0.82) and 1.78 (95% CI: 1.46, 2.16), respectively] (**Table 4**). After adjusting for conventional factors accountable for initiating KRT, higher CMPF remained independently associated with reduced risk of KRT (HR: 0.82; 95% CI: 0.73, 0.91; model 1). The significant association between higher CMPF and lower risk of KRT withstood further extensive adjustment in model 3 (HR: 0.80; 95% CI: 0.71, 0.90). No significant association between TMAO and risk of KRT was observed in adjusted models.

**TABLE 4:** Association between TMAO, CMPF, and risk of kidney replacement therapy^1^

	KRT initiation, HR [95%CI]	
	ln CMPF	ln TMAO
Crude	0.74 [0.67,0.82]	1.78 [1.46,2.16]
Model 1	0.82 [0.73,0.91]	1.11 [0.88,1.40]
Model 2	0.79 [0.71,0.89]	1.04 [0.83,1.31]
Model 3	0.80 [0.71,0.90]	1.01 [0.81,1.27]

1 *n* = 737. Crude model: ln TMAO + ln CMPF. Model 1: adjusted for age, sex, eGFR, and ACR. Model 2: model 1 + country, diabetes, preexisting cardiovascular disease, Charlson comorbidity index, diastolic blood pressure, systolic blood pressure, and malnutrition (SGA <5). Model 3: model 2 + albumin, phosphate, and hemoglobin. ACR, albumin creatinine ratio; CMPF, 3-carboxy-4-methyl-5-propyl-2-furanpropionate; eGFR, estimated glomerular filtration rate; KRT, kidney replacement therapy; SGA, subjective global assessment; TMAO, trimethylamine N-oxide.

**FIGURE 2:**
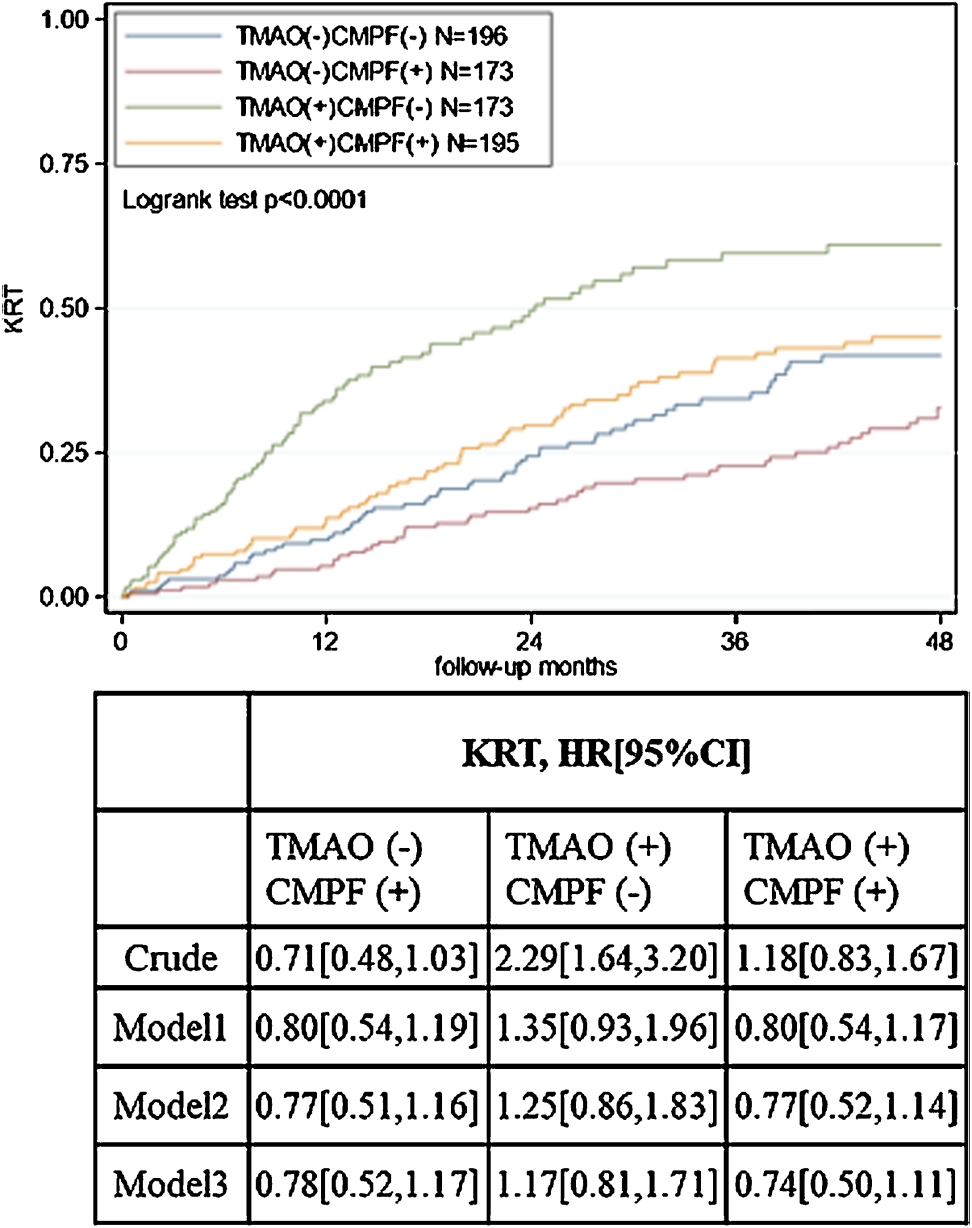
Kaplan–Meier estimates for risk of KRT initiation and cause-specific Cox regression models according to TMAO and CMPF median groups. Model 1: adjusted for age, sex, eGFR, ACR. Model 2: adjusted as for model 1 plus country, diabetes, preexisting cardiovascular disease, Charlson comorbidity index, diastolic blood pressure, systolic blood pressure, malnutrition (SGA <5). Model 3: adjusted as for model 2 plus albumin, phosphate, hemoglobin. ACR, albumin creatinine ratio; CMPF, 3-carboxy-4-methyl-5-propyl-2-furanpropionate; eGFR, estimated glomerular filtration rate; KRT, kidney replacement therapy; SGA, subjective global assessment; TMAO, trimethylamine N-oxide.

We next looked at the risk of KRT stratified by our predefined subgroups of TMAO and CMPF (**Figure 2**). In the crude model, compared with the reference group, patients with TMAO (+) CMPF (–) had a significantly increased risk of KRT initiation (HR: 2.29; 95% CI: 1.64, 3.20). Compared with the reference group, no significant associations between TMAO (–) CMPF (+) group nor TMAO (+) CMPF (+) group and risk of KRT initiation were observed in a crude model and models adjusted for other confounders.

#### TMAO, CMPF, and first MACE

We also explored the association between TMAO, CMPF, and first MACE. High TMAO was associated with increased risk of first MACE in crude models and models adjusted for several confounders (26–34% higher risk) but not in model 4 further adjusted for eGFR. In contrast, CMPF was not associated with the risk of first MACE (**Supplemental Table 3**). Compared with the reference group [TMAO (–) CMPF (–)], TMAO- and CMPF-stratified groups were not associated with the risk of first MACE (**Supplemental Table 4**).

## Discussion

In this large, prospective, multicenter study of older adults (≥65 y) with CKD stage 4–5 across several European countries, there are several key findings. First, whereas higher TMAO is associated with increased risk of all-cause mortality, higher CMPF is independently associated with reduced risk of all-cause mortality and KRT initiation. Second, we found that, compared with a reference group with low concentrations of both TMAO and CMPF, patients with low TMAO and high CMPF concentrations portend a robust survival benefit in reducing all-cause mortality; in addition, patients with high TMAO and high CMPF do not demonstrate an increased risk of all-cause mortality and KRT. Our findings support the hypothesis the high circulating CMPF conferred an independent role in health benefits and might even counteract the unfavorable association of TMAO with outcomes.

The role of TMAO in mediating CV events has been fairly recognized across various populations (14), but the magnitude remains ambiguous. A study in mixed-race healthy US subjects reported that TMAO was not associated with atherosclerosis during a 10-y follow-up (30). Similarly, several observational follow-up studies failed to confirm the association between TMAO and CV outcomes (31, 32). In CKD, elevated circulating TMAO concentrations were well documented, with a large variability among studies (11, 13, 16, 17, 33, 34). Here we report >5-fold higher TMAO concentrations (median: 18.4; IQR: 12.0–29.2 µM) compared with a healthy population (33) (median: 3.3; IQR: 3.1–6.0 µM). We found that high TMAO conferred a 46–63% higher risk of all-cause mortality. However, the association between high TMAO and high risk of mortality was likely to be counteracted with a high dietary fish intake, suggested by concomitant high CMPF concentrations. Although TMAO is commonly recognized as a microbiota-dependent metabolite of mainly red meat/egg consumption (35), it is also an indicative metabolite of fish intake (36). Nevertheless, TMAO alone had a poor ability to estimate fish intake (37), largely due to the undetermined concentration across fish products habitating at different depths (36). In contrast, CMPF is considered a prominent metabolite following the consumption of fish oil and diets rich in fish (20, 23). The correlation between plasma, serum, and urine CMPF and habitual fish/shellfish intake has been verified in several populations (38–40).

Here we found that high CMPF was consistently associated with reduced risk of all-cause mortality and KRT initiation. This observation was counterintuitive to the concept of CMPF as a uremic toxin that can cause damage to proximal tubular cells, at least in vitro (41). To date, there is insufficient evidence of the impact of CMPF on human health. In vivo and in vitro studies from Prentice et al. (42) and Liu et al. (43) showed that CMPF can impair B-cell function, mitochondrial function, and glucose metabolism. In contrast, CMPF was reported to be able to regulate hepatic lipid homeostasis and reverse steatosis (20, 44). Moreover, high CMPF was not associated with all-cause and CV mortality in dialysis patients (45). Taken together, the knowledge of the role of CMPF in human health is still in its infancy and future investigations are warranted to fill the knowledge gap of such conflicting observations.

We hypothesized that overall high fish CMPF (+) and low red meat TMAO (–) consumption can predispose to health benefits in CKD. Our results support this hypothesis in finding that patients with low TMAO and high CMPF had a lower risk of all-cause mortality, whereas high concomitant CMPF and TMAO did not show increased risk of death. Hence, we posit that high CMPF, representing high fish intake, is associated with overall better clinical outcomes regardless of TMAO concentrations. Such postulation of CMPF-related health benefits corroborates with analysis when CMPF was treated as a continuous variable, where high CMPF was significantly associated with a lower risk of all-cause mortality and KRT independently of TMAO. Indeed, the paradox of TMAO as a friend or foe can be partially justified by a background diet pattern as it was recently found that a “healthy diet” rich in fish or in whole-grain cereals, but not meat, processed meat, or dairy products, significantly increased plasma TMAO (46).

We acknowledge the strengths and limitations of this study. A major strength is the well-phenotyped inception cohort of CKD stage 4–5 patients across Europe with long-term follow-up. Our study is, however, limited by its observational study design and, as such, we cannot conclude the causal effect of TMAO and CMPF on clinical outcomes. Also, as TMAO and CMPF are metabolites derived from dietary sources with possible involvement of host gut microbial activities, the lack of information on dietary pattern and gut microbiome profile is a vital limitation. It is worth noting, however, that patients recruited in Sweden had significantly higher CMPF concentrations. Despite the lack of actual diet evaluation, this probably mirrors the food culture of an overall high fish and seafood consumption in the Scandinavian region. In addition, although we have corrected eGFR, this does not account for tubular function, which can be highly variable at any given eGFR (34). Hence, the independent associations between TMAO, CMPF, and outcomes need to be interpreted with caution.

In summary, in our investigation of the association between TMAO and CMPF and their combinations with clinical outcomes in older CKD 4–5 patients, we observed a strong beneficial profile of high circulating CMPF and its association with reduced risk of all-cause mortality and KRT initiation independently of TMAO concentrations. While the dietary source and the metabolic profile of TMAO remain to be elucidated, our results suggest that high CMPF confers a beneficial role in CKD, possibly due to a healthy eating habit of fish consumption. Moreover, it may possibly counteract the unfavorable association between TMAO and adverse outcomes. As such, whether CMPF is a marker or protective factor, it offers the potential to be a modifiable and important target to improve outcomes in CKD in future studies.

## Supplementary Material

nqac278_Supplemental_File

## Data Availability

Data described in the manuscript, code book, and analytic code will be made available upon reasonable request to the corresponding author. Please e-mail marie.evans@ki.se.

## Notes

The main funding was received from the European Renal Association (ERA) and contributions from the Swedish Medical Association (SLS), the Stockholm County Council ALF Medicine and Center for Innovative Research (CIMED), the Italian Society of Nephrology (SIN-Reni), the Dutch Kidney Foundation (SB 142), the Young Investigators grant in Germany, and the National Institute for Health Research (NIHR) in the United Kingdom.

Supplemental Figure 1 and Supplemental Tables 1–4 are available from the “Supplementary data” link in the online posting of the article and from the same link in the online table of contents at https://academic.oup.com/ajcn/.

Abbreviations used: ACR, albumin creatinine ratio; BP, blood pressure; CKD, chronic kidney disease; CMPF, 3-carboxy-4-methyl-5-propyl-2-furanpropionate; CV, cardiovascular; CVD, cardiovascular disease; eGFR, estimated glomerular filtration rate; EQUAL, European QUALity (study); KRT, kidney replacement therapy; MACE, major adverse cardiac event; SGA, subjective global assessment; TMAO, trimethylamine N-oxide.
