# Supplementary material for: The association between TMAO, CMPF, and clinical outcomes in advanced chronic kidney disease: results from the European QUALity (EQUAL) Study
Source: Am J Clin Nutr. 2022 Sep 27;116(6):1842–51. doi: 10.1093/ajcn/nqac278 (PMC9761748; doi:10.1093/ajcn/nqac278)
Supplement: nqac278_Supplemental_File [file nqac278_supplemental_file.docx]

**SUPPLEMENT**

**The association between TMAO, CMPF and clinical outcomes in advanced CKD; results from the EQUAL study**

Lu Dai, Ziad A. Massy, Peter Stenvinkel, Nicholas C Chesnaye, Islam Amine Larabi, Jean Claude Alvarez, Fergus J Caskey, Claudia Torino, Gaetana Porto, Maciej Szymczak, Magdalena Krajewska, Christiane Drechsler, Christoph Wanner, Kitty J. Jager, Friedo W. Dekker, Pieter Evenepoel, Marie Evans, and the EQUAL study investigators

**Supplementary Figure 1.**


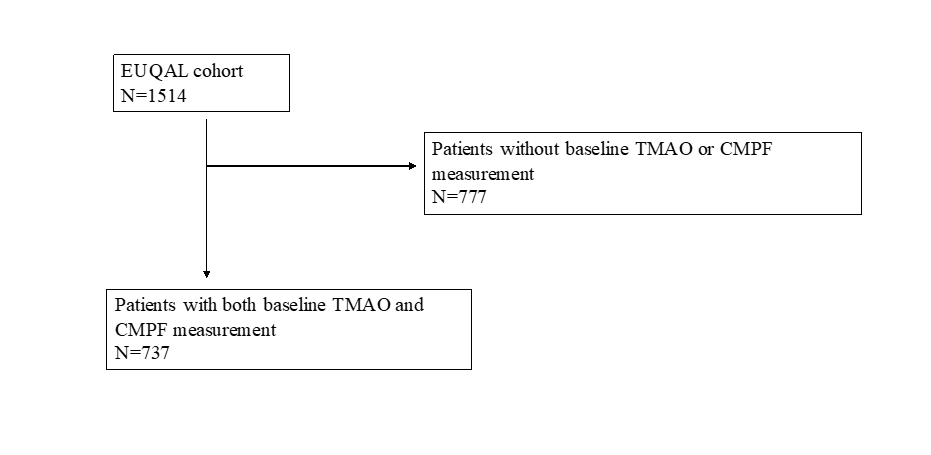


**Supplementary Table 1. Baseline characteristics according to tertiles of TMAO (N=737)**

|  | **Lower Tertile** | **Middle tertile** | **Higher tertile** | **p-value** |
| --- | --- | --- | --- | --- |
|  | **N=246** | **N=246** | **N=245** |  |
| Age (years) | 76.4 (70.2, 81.4) | 76.2 (70.5, 80.8) | 75.7 (70.4, 80.5) | 0.73 |
| Male sex (n, %) | 131 (53.3%) | 157 (63.8%) | 177 (72.2%) | <0.001 |
| Country |  |  |  | 0.05 |
| Germany | 34 (13.8%) | 48 (19.5%) | 33 (13.5%) |  |
| Poland | 17 (6.9%) | 25 (10.2%) | 19 (7.8%) |  |
| Sweden | 61 (24.8%) | 73 (29.7%) | 79 (32.2%) |  |
| UK | 134 (54.5%) | 100 (40.7%) | 114 (46.5%) |  |
| Primary renal diagnosis (n, %) (n=245/242/242) |  |  |  | 0.48 |
| Glomerular disease | 34 (13.8%) | 24 (9.8%) | 27 (11.0%) |  |
| Tubulo-interstitial disease | 23 (9.3%) | 18 (7.3%) | 22 (9.0%) |  |
| Systemic disease affecting the kidney | 7 (2.8%) | 6 (2.4%) | 8 (3.3%) |  |
| Diabetes | 41 (16.7%) | 61 (24.8%) | 63 (25.7%) |  |
| Hypertension | 81 (32.9%) | 77 (31.3%) | 74 (30.2%) |  |
| Familial/Hereditary nephropathies | 10 (4.1%) | 15 (6.1%) | 8 (3.3%) |  |
| Miscellaneous renal disorders | 11 (4.5%) | 5 (2.0%) | 7 (2.9%) |  |
| Unknown | 38 (15.4%) | 36 (14.6%) | 33 (13.5%) |  |
| Charlson comorbidity index (n=244/241/241) | 7 (5, 8) | 7 (6, 8) | 7 (6, 8) | 0.07 |
| Previous CVD (n, %) | 104 (42.3%) | 116 (47.2%) | 115 (46.9%) | 0.42 |
| Diabetes (n, %) | 77 (31.3%) | 116 (47.2%) | 126 (51.4%) | <0.001 |
| eGFR (MDRD, ml/min/1.73m^2^) | 21.1 (18.8, 24.7) | 19.5 (16.1, 22.6) | 17.5 (13.4, 21.3) | <0.001 |
| Diastolic BP (mmHg) (n=243/244/240) | 74 (67, 81) | 75 (68, 83) | 75 (65, 82) | 0.61 |
| Systolic BP (mmHg) (n=243/244/240) | 146 (131, 161) | 144 (130, 160) | 150 (134, 162) | 0.18 |
| Height (cm) (n=235/235/225) | 166 (159, 174) | 170 (161, 176) | 170 (163, 175) | <0.001 |
| Weight (kg) (n=235/240/237) | 77.2 (68.2, 91.1) | 80.1 (71.4, 90.6) | 80.5 (70.0, 93.0) | 0.11 |
| BMI (kg/m^2^) (n=228/234/224) | 28.2 (24.8, 31.9) | 28.3 (25.1, 32.1) | 27.8 (24.8, 32.1) | 0.91 |
| BMI group (kg/m^2^) (n=228/234/224) |  |  |  | 0.97 |
| <22 | 15 (6.1%) | 17 (6.9%) | 17 (6.9%) |  |
| 22-24.9 | 46 (18.7%) | 41 (16.7%) | 43 (17.6%) |  |
| 25-29.9 | 78 (31.7%) | 89 (36.2%) | 83 (33.9%) |  |
| >30 | 89 (36.2%) | 87 (35.4%) | 81 (33.1%) |  |
| Waist circumference (cm) (n=234/235/231) | 103 (93 , 113) | 104 (96, 113) | 102 (96, 114) | 0.71 |
| Alpha-blocker (n, %) | 60 (24.4%) | 63 (25.6%) | 81 (33.1%) | 0.07 |
| Beta-blocker (n, %) | 124 (50.4%) | 143 (58.1%) | 147 (60.0%) | 0.08 |
| ACEi/ARB (n, %) | 130 (52.8%) | 132 (53.7%) | 123 (50.2%) | 0.73 |
| Lipid-lowering (n, %) | 160 (65.0%) | 160 (65.0%) | 146 (59.6%) | 0.35 |
| SGA overall assessment | 6.0 (5.0, 7.0) | 6.0 (5.0, 7.0) | 6.0 (5.0, 7.0) | 0.68 |
| SGA<5 (malnourished) | 73 (29.7%) | 80 (32.5%) | 86 (35.1%) | 0.44 |
| Low protein diet prescription  (n, %) (n=246/244/242) | 15 (6.1%) | 31 (12.7%) | 22 (9.1%) | 0.04 |
| Hemoglobin (mmol/L) (n=238/239/238) | 7.3 (6.7, 7.9) | 7.2 (6.6, 7.9) | 7.0 (6.4, 7.8) | 0.01 |
| Sodium (mmol/L) (n=242/241/238) | 140.0 (138.0, 142.0) | 141.0 (139.0, 142.0) | 141.0 (139.0, 142.0) | 0.90 |
| Potassium (mmol/L) (n=242/242/240) | 4.6 (4.2, 4.9) | 4.6 (4.2, 5.0) | 4.7 (4.2, 5.1) | 0.44 |
| Calcium (mmol/L) (n=234/230/235) | 2.3 (2.2, 2.4) | 2.3 (2.1, 2.4) | 2.2 (2.1, 2.4) | <0.001 |
| Phosphate (mmol/L) (n=228/228/234) | 1.2 (1.1, 1.4) | 1.3 (1.1, 1.4) | 1.3 (1.1, 1.5) | <0.001 |
| Urea (mmol/L) (n=241/237/236) | 16.1 (13.2, 19.2) | 17.5 (14.8, 22.5) | 20.4 (16.8, 24.8) | <0.001 |
| Albumin (g/L) (n=223/213/221) | 38.0 (36.0, 42.0) | 38.0 (34.0, 41.0) | 37.0 (34.0, 41.0) | 0.01 |
| Total cholesterol (mmol/L) (n=185/186/179) | 4.6 (3.9, 5.5) | 4.4 (3.6, 5.5) | 4.6 (3.7, 5.3) | 0.49 |
| PTH (pmol/L) (n=193/197/197) | 13.1 (8.1, 19.4) | 15.2 (8.2, 24.3) | 18.0 (12.4, 29.4) | <0.001 |
| ACR (mg/mmol) (n=101/113/121) | 38.0 (5.6, 125.0) | 58.9 (13.1, 219.0) | 61.5 (11.3, 266.2) | 0.08 |
| TMAO (µM) | 10.2 (7.1, 11.9) | 18.2 (15.9, 21.0) | 37.4 (28.5, 47.6) | <0.001 |
| CMPF (µM) | 5.3 (2.2, 13.2) | 8.3 (3.2, 15.7) | 7.7 (2.7, 15.4) | 0.01 |

Data are presented as median (interquantile range IQR), mean with standard deviation (SD), or number (%) as appropriate.

Abbreviations: TMAO, trimethylamine N-oxide; CVD, cardiovascular disease; eGFR, estimated glomerular filtration rate; MDRD, the Modification of Diet in Renal Disease; BP, blood pressure; BMI, body mass index; ACEi/ARB, angiotensin-converting enzyme inhibitor/angiotensin receptor blocker; SGA, subjective global assessment; PTH, parathyroid hormone; ACR, albumin creatinine ratio; CMPF, 3-carboxy-4-methyl-5-propyl-2-furanpropionate

**Supplementary Table** **2. Baseline characteristics according to tertiles of CMPF(N=737)**

|  | **Lower Tertile** | **Middle tertile** | **Higher tertile** | **p-value** |
| --- | --- | --- | --- | --- |
|  | **N=246** | **N=246** | **N=245** |  |
| Age (years) | 74.4 (69.5, 79.8) | 76.0 (70.2, 80.3) | 77.3 (71.1, 82.2) | 0.005 |
| Male sex (n, %) | 145 (58.9%) | 159 (64.6%) | 161 (65.7%) | 0.25 |
| Country |  |  |  | <0.001 |
| Germany | 35 (14.2%) | 49 (19.9%) | 31 (12.7%) |  |
| Poland | 20 (8.1%) | 23 (9.3%) | 18 (7.3%) |  |
| Sweden | 36 (14.6%) | 63 (25.6%) | 114 (46.5%) |  |
| UK | 155 (63.0%) | 111 (45.1%) | 82 (33.5%) |  |
| Primary renal diagnosis (n, %) (n=242/243/244) |  |  |  | 0.008 |
| Glomerular disease | 33 (13.4%) | 36 (14.6%) | 16 (6.5%) |  |
| Tubulo-interstitial disease | 18 (7.3%) | 26 (10.6%) | 19 (7.8%) |  |
| Systemic disease affecting the kidney | 10 (4.1%) | 6 (2.4%) | 5 (2.0%) |  |
| Diabetes | 64 (26.0%) | 47 (19.1%) | 54 (22.0%) |  |
| Hypertension | 65 (26.4%) | 71 (28.9%) | 96 (39.2%) |  |
| Familial/Hereditary nephropathies | 5 (2.0%) | 12 (4.9%) | 16 (6.5%) |  |
| Miscellaneous renal disorders | 6 (2.4%) | 10 (4.1%) | 7 (2.9%) |  |
| Unknown | 41 (16.7%) | 35 (14.2%) | 31 (12.7%) |  |
| Charlson comorbidity index (n=240/242/244) | 7 (6, 8) | 7 (6, 8) | 7 (6, 8) | 0.95 |
| Previous CVD (n, %) | 109 (44.3%) | 122 (49.6%) | 104 (42.4%) | 0.17 |
| Diabetes (n, %) | 120 (48.8%) | 98 (39.8%) | 101 (41.2%) | 0.08 |
| eGFR (MDRD, ml/min/1.73m^2^) | 19.4 (15.4, 23.0) | 19.7 (15.4, 23.1) | 19.7 (16.8, 22.9) | 0.37 |
| Diastolic BP (mmHg) (n=242/241/244) | 72 (66, 80) | 75 (68, 83) | 76 (67, 82) | 0.04 |
| Systolic BP (mmHg) (n=242/241/244) | 147 (131, 162) | 145 (132, 160) | 148 (132, 160) | 0.89 |
| Height (cm) (n=228/229/238) | 168 (160, 175) | 169 (161, 175) | 170 (163, 175) | 0.15 |
| Weight (kg) (n=236/234/242) | 80.3 (69.7, 93.5) | 79.0 (69.1, 87.8) | 80.3 (70.0, 91.2) | 0.40 |
| BMI (kg/m^2^) (n=225/225/236) | 28.6 (25.3, 32.4) | 27.5 (24.5, 31.8) | 28.2 (24.9, 31.7) | 0.16 |
| BMI group (kg/m^2^) (n=225/225/236) |  |  |  | 0.31 |
| <22 | 19 (7.7%) | 13 (5.3%) | 17 (6.9%) |  |
| 22-24.9 | 35 (14.2%) | 50 (20.3%) | 45 (18.4%) |  |
| 25-29.9 | 78 (31.7%) | 89 (36.2%) | 83 (33.9%) |  |
| >30 | 93 (37.8%) | 73 (29.7%) | 91 (37.1%) |  |
| Waist circumference (cm) (n=235/238/227) | 106 (95, 115) | 101 (95 , 111) | 103 (95, 114) | 0.08 |
| Alpha-blocker (n, %) | 82 (33.3%) | 64 (26.0%) | 58 (23.7%) | 0.04 |
| Beta-blocker (n, %) | 139 (56.5%) | 134 (54.5%) | 141 (57.6%) | 0.78 |
| ACEi/ARB (n, %) | 118 (48.0%) | 126 (51.2%) | 141 (57.6%) | 0.10 |
| Lipid-lowering (n, %) | 180 (73.2%) | 150 (61.0%) | 136 (55.5%) | <0.001 |
| SGA overall assessment | 6.0 (5.0, 6.0) | 6.0 (5.0, 7.0) | 6.0 (5.0, 7.0) | 0.02 |
| SGA<5 (malnourished) | 88 (35.8%) | 72 (29.3%) | 79 (32.2%) | 0.30 |
| Low protein diet prescription  (n, %) (n=243/244/245) | 13 (5.3%) | 19 (7.8%) | 36 (14.7%) | 0.001 |
| Hemoglobin (mmol/L) (n=240/236/239) | 7.0 (6.5, 7.7) | 7.2 (6.7, 7.9) | 7.3 (6.7, 7.9) | 0.003 |
| Sodium (mmol/L) (n=241/238/242) | 140.0 (138.0, 142.0) | 141.0 (139.0, 142.0) | 141.0 (139.0, 143.0) | 0.57 |
| Potassium (mmol/L) (n=240/242/242) | 4.7 (4.2, 5.0) | 4.6 (4.2, 5.0) | 4.6 (4.2, 5.0) | 0.27 |
| Calcium (mmol/L) (n=229/230/240) | 2.3 (2.1, 2.4) | 2.3 (2.2, 2.4) | 2.3 (2.2, 2.4) | 0.07 |
| Phosphate (mmol/L) (n=225/229/236) | 1.3 (1.1, 1.5) | 1.3 (1.1, 1.4) | 1.2 (1.1, 1.4) | 0.02 |
| Urea (mmol/L) (n=239/236/239) | 17.7 (14.9, 21.7) | 17.5 (14.6, 22.6) | 18.1 (14.5, 22.5) | 0.91 |
| Albumin (g/L) (n=221/208/228) | 38.0 (34.0, 41.0) | 37.0 (34.0, 41.3) | 38.0 (35.0, 41.0) | 0.69 |
| Total cholesterol (mmol/L) (n=172/175/203) | 4.2 (3.5, 5.2) | 4.9 (3.9, 5.6) | 4.5 (3.8, 5.6) | 0.004 |
| PTH (pmol/L) (n=193/187/207) | 13.8 (8.0, 23.5) | 16.3 (9.9, 25.8) | 15.9 (9.9, 22.5) | 0.12 |
| ACR (mg/mmol) (n=89/108/138) | 128.4 (13.5, 369.6) | 79.4 (9.0, 216.9) | 25.1 (5.9, 85.8) | <0.001 |
| TMAO (µM) | 16.7 (10.4, 26.1) | 19.6 (12.6, 28.9) | 18.2 (13.3, 31.4) | 0.01 |
| CMPF (µM) | 1.8 (0.8, 2.6) | 7.2 (5.3, 9.1) | 18.9 (14.9, 24.8) | <0.001 |

Data are presented as median (interquantile range IQR), mean with standard deviation (SD), or number (%) as appropriate.

Abbreviations: CMPF, 3-carboxy-4-methyl-5-propyl-2-furanpropionate; CVD, cardiovascular disease; eGFR, estimated glomerular filtration rate; MDRD, the Modification of Diet in Renal Disease; BP, blood pressure; BMI, body mass index; ACEi/ARB, angiotensin-converting enzyme inhibitor/angiotensin receptor blocker; SGA, subjective global assessment; PTH, parathyroid hormone; ACR, albumin creatinine ratio; TMAO, trimethylamine N-oxide

**Supplementary Table 3. Association between TMAO, CMPF and risk of first MACE (N=737)**

|  | **Risk of first MACE, HR[95%CI]** | |
| --- | --- | --- |
|  | lnCMPF | lnTMAO |
| Crude | 0.94[0.84,1.04] | 1.34[1.08,1.65] |
| Model1 | 0.87[0.77,0.98] | 1.32[1.07,1.64] |
| Model2 | 0.90[0.79,1.01] | 1.28[1.03,1.59] |
| Model3 | 0.92[0.81,1.03] | 1.26[1.00,1.57] |
| Model4 | 0.91[0.81,1.04] | 1.24[0.98,1.57] |

Crude model: lnTMAO+ lnCMPF

Model1, adjusted for age, sex, country

Model2, model1 + DM, CVD, Charlson comorbidity index, diastolic BP, systolic BP, SGA

Model3, model2 + albumin, phosphate, hemoglobin

Model4, model3 + eGFR

**Supplementary Table 4. Association between TMAO and CMPF subgroups with risk of first MACE(N=737)**

|  | | **Risk of first MACE, HR[95%CI]** | | |
| --- | --- | --- | --- | --- |
|  | TMAO (-) CMPF (+) | TMAO (+) CMPF (-) | TMAO (+) CMPF (+) |  |
| Crude | 0.92[0.63, 1.35] | 1.26[0.87,1.83] | 0.96[0.66,1.41] |  |
| Model1 | 0.81[0.55,1.20] | 1.23[0.85,1.79] | 0.82[0.55,1.22] |  |
| Model2 | 0.89[0.60,1.32] | 1.23[0.85,1.79] | 0.86[0.57,1.28] |  |
| Model3 | 0.93[0.63,1.38] | 1.21[0.82,1.78] | 0.86[0.57,1.28] |  |
| Model4 | 0.93[0.63,1.39] | 1.16[0.79,1.73] | 0.83[0.55,1.25] |  |

Model1, adjusted for age, sex, country

Model2, model1 + DM, CVD, Charlson comorbidity index, diastolic BP, systolic BP, SGA

Model3, model2 + albumin, phosphate, hemoglobin

Model4, model3 + eGFR
